# TOMATOMICS: A Web Database for Integrated Omics Information in Tomato

**DOI:** 10.1093/pcp/pcw207

**Published:** 2017-01-06

**Authors:** Toru Kudo, Masaaki Kobayashi, Shin Terashima, Minami Katayama, Soichi Ozaki, Maasa Kanno, Misa Saito, Koji Yokoyama, Hajime Ohyanagi, Koh Aoki, Yasutaka Kubo, Kentaro Yano

**Affiliations:** 1 Bioinformatics Laboratory, School of Agriculture, Meiji University, 1-1-1 Higashi-mita, Tama-ku, Kawasaki, Kanagawa 214-8571, Japan.; 2 King Abdullah University of Science and Technology (KAUST), Computational Bioscience Research Center (CBRC), Thuwal 23955-6900, Kingdom of Saudi Arabia.; 3 Graduate School of Life and Environmental Sciences, Osaka Prefecture University, 1-1 Gakuen-cho, Naka-ku, Sakai, 599-8531 Japan.; 4 Graduate School of Environmental and Life Science, Okayama University, Okayama, 700-8530 Japan

**Keywords:** Database, Genome, Micro-Tom, *Solanum lycopersicum* (tomato), Transcriptome

## Abstract

*Solanum lycopersicum* (tomato) is an important agronomic crop and a major model fruit-producing plant. To facilitate basic and applied research, comprehensive experimental resources and omics information on tomato are available following their development. Mutant lines and cDNA clones from a dwarf cultivar, Micro-Tom, are two of these genetic resources. Large-scale sequencing data for ESTs and full-length cDNAs from Micro-Tom continue to be gathered. In conjunction with information on the reference genome sequence of another cultivar, Heinz 1706, the Micro-Tom experimental resources have facilitated comprehensive functional analyses. To enhance the efficiency of acquiring omics information for tomato biology, we have integrated the information on the Micro-Tom experimental resources and the Heinz 1706 genome sequence. We have also inferred gene structure by comparison of sequences between the genome of Heinz 1706 and the transcriptome, which are comprised of Micro-Tom full-length cDNAs and Heinz 1706 RNA-seq data stored in the KaFTom and Sequence Read Archive databases. In order to provide large-scale omics information with streamlined connectivity we have developed and maintain a web database TOMATOMICS (http://bioinf.mind.meiji.ac.jp/tomatomics/). In TOMATOMICS, access to the information on the cDNA clone resources, full-length mRNA sequences, gene structures, expression profiles and functional annotations of genes is available through search functions and the genome browser, which has an intuitive graphical interface.

## Introduction

*Solanum lycopersicum* (tomato) is a model plant for studies both of plants yielding berry-type fruit and of the Solanaceae (Mueller et al. 2005, Samuels 2015). To facilitate biological studies using tomato, databases provide diverse omics information for research communities. For example, the Sol Genomics Network (SGN) (Fernandez-Pozo et al. 2015) provides information on the genome sequence of cultivar Heinz 1706 and structural and functional annotation of the genome (Tomato Genome Consortium 2012, Fernandez-Pozo et al. 2015). The MiBASE database (Yano et al. 2006) stores information on 125,883 ESTs from the miniature tomato cultivar Micro-Tom (Scott and Harbaugh 1989, Meissner et al. 1997) and unigenes (a non-redundant sequence set of expressed genes) deduced from assemblies of publicly available tomato expressed sequence tags (ESTs). Information on 13,227 full-length cDNAs (named high-throughput cDNA sequences or HTCs) generated from Micro-Tom and their functional annotations are accessible from the KaFTom database (Aoki et al. 2010). In addition, the TOMATOMA database (Shikata et al. 2016) provides information on mutant resources established in a Micro-Tom genomic background. Major omics databases providing large-scale omics information have increased studies of the Solanaceae family including tomato, and vice versa.

An integrated web-based framework allowing easy access to all the multi-omics resources in tomato is promising for rapid and efficient advances in Solanaceae biology. By integrating omics information generated from model cultivars Heinz 1706 and Micro-Tom, streamlined access and interconnection of multiple independent sources of information is achievable. In addition, the comparative approach of multi-omics information between Heinz 1706 and Micro-Tom will bring new insights and better evidence to structural and functional annotation of the tomato genome. In order to achieve this we have constructed, and maintain, the TOMATOMICS database (http://bioinf.mind.meiji.ac.jp/tomatomics/) providing multi-omics information in tomato.

Here, we introduce the multi-omics information and database functions of the database. TOMATOMICS contains omics information such as genome sequences, genome annotations, transcriptome sequences, amino acid sequences, and single nucleotide polymorphisms (SNPs). With the aim of integrating multiple resources seamlessly, we established a nomenclature for tomato locus identifiers (IDs), named TMCS, based both on the genome sequences themselves and RNA-seq expression evidence. It contains loci, un-translated regions (UTRs) and splicing variants never previously described. Simultaneously, IDs for locus groups were defined for convenience in searching for correspondence among transcript sequences provided from different sources. Each locus group involves ESTs, HTCs and transcripts predicted in the genome that potentially originated from each single locus. With these new IDs for loci and locus groups, the information on the genome and transcripts is easily and quickly accessible in TOMATOMICS. TOMATOMICS provides powerful database functions for searching, browsing, retrieving, visualizing, and downloading information through a simple, intuitive and interactive graphical web interface.

## Results

### Integration of transcriptome resources and novel prediction of gene structures

Ordering cDNA and EST clones is the usual way to obtain polynucleotides derived from a gene targeted in a study. Thus, information on correspondence between ESTs and HTCs, and on annotated gene models is crucial to promote research using molecular tools. Moreover, such information may suggest new gene loci and transcript variants, advancing understanding of genome systems and gene functions. In this study, bioinformatics analyses were performed to reveal the correspondence between the existing transcriptome sequences and structural annotations of tomato genes (ITAG2.4) predicted by the International Tomato Annotation Group (ITAG) (Fernandez-Pozo et al. 2015). In addition, to expand the usefulness of available transcriptome information, gene structures were predicted using RNA-seq transcriptome data.

#### Mapping of transcriptome sequences to the reference genome

In order to determine expression evidence for gene structure predictions, firstly, all transcript sequences were mapped to the Heinz 1706 reference genome SL2.50 (Fernandez-Pozo et al. 2015) so that all transcript sequences are directly assigned according to their genomic positions. TOMATOMICS currently stores 300,541, 13,150, and 42,257 sequences of ESTs, HTCs, and SGN unigenes, respectively. Among them, 292,325 ESTs, 12,986 HTCs, and 40,614 unigenes were mapped to the reference genome but 8,216 ESTs, 164 HTCs, and 1,643 unigenes were not. Whereas we can speculate that the unmapped sequences are a mixture of experimental or computational artifacts and transcripts derived from genes that exist on the Micro-Tom genome but not on the Heinz 1706 genome, it is hard to conclude this issue unless genome sequence of Micro-Tom is determined.

In addition, RNA-derived short reads obtained by RNA-seq analysis of leaves, roots, flowers, flower buds, and fruit during ripening of Heinz 1706 and of flowers before and after anthesis of Micro-Tom were collected from the Sequence Read Archive (SRA; Kodama et al. 2012), then mapped to the reference genome. In total, approximately 340 million sequencing reads were mapped to the reference genome after trimming of adaptor sequences and filtering out low-quality bases. Statistics of the read preprocessing and mapping are summarized in Supplementary Table S1. By mapping the transcript sequences to the reference genome sequences, these sequences can now be easily compared with ITAG2.4 gene structures (Fernandez-Pozo et al. 2015); the mapped results can be visualized in a genome browser, JBrowse, as explained later.

As resources of Micro-Tom transcripts, EST and HTC clones are available from the National BioResource Project (NBRP) Tomato (http://tomato.nbrp.jp/indexEn.html). In TOMATOMICS external hyperlinks to the portal page for the clone requests are on the information pages of the Micro-Tom ESTs and HTCs.

#### Prediction of gene structures of tomato and establishment of TMCS locus and transcript ID nomenclature

We predicted gene structures in the tomato genome according to the RNA-seq reads of Heinz 1706 mapped on the reference genome. The SRA database stores tomato RNA-seq data not only from Heinz 1706 but also from different cultivars including Micro-Tom. Whereas the sequencing data from the other cultivars might lead to prediction of more genes, they may wrongly predict gene structures due to DNA polymorphisms between cultivars. To avoid the problem, we only employed the RNA-seq reads from Heinz 1706 for the gene structure analysis. This gene structure analysis resulted in 28,796 loci and 54,783 transcripts predicted in the tomato genome.

This genome annotation was termed TMCS (version 1.2.1), a shortened version of TOMATOMICS. We created locus and transcript IDs for TMCS v1.2.1, employing a nomenclature which is similar to that used in the rice and Arabidopsis genome sequencing projects (Sakai et al. 2013, Berardini et al. 2015). Explaining with an example, the locus ID TMCS02g1018880 is comprised of the prefix ‘TMCS’, two digits (02) indicating the chromosome number, the letter ‘g’ meaning a gene, and seven digits, which are a number serially assigned to each locus within a chromosome. The transcript ID TMCS02g1018880-01 appeared by adding a hyphen and two digits for each splicing variant at the ID of the locus from which the transcript is transcribed. Among the TMCS loci, 415 were not predicted by ITAG2.4 or HTC mapping (Supplementary Table S2).

#### Characterization of the TMCS genome annotation

**Fig. 1:**
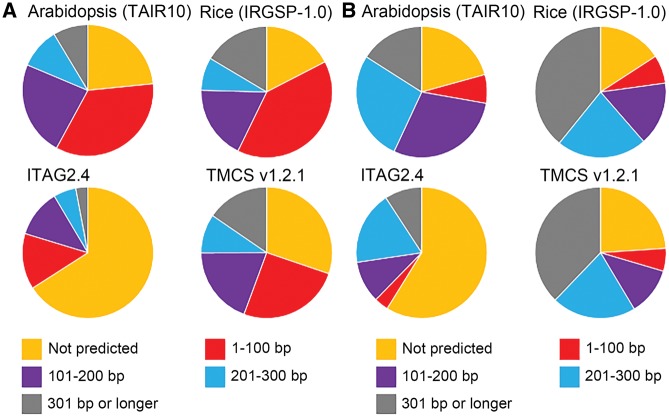
Transcripts with predicted 5′-UTRs and 3′-UTR. Frequency distribution of 5′-UTR (A) and 3′-UTR (B) lengths in the genomes of Arabidopsis (TAIR10), rice (IRGSP-1.0) and tomato (ITAG2.4 and TMCS v1.2.1). TMCS v1.2.1 has the gene structural annotations obtained from this study.

Of the 54,783 TMCS transcripts, we predicted 5′-UTRs in 38,228 transcripts but not in 16,555 transcripts, and 3′-UTRs in 41,657 but not in 13,126 transcripts (Supplementary Fig. S1). The proportions of transcripts lacking the predicted 5′-UTR (approx. 30%) and 3′-UTR (approximately 24%) were comparable to those in the genome annotations of Arabidopsis (TAIR10; Lamesch et al. 2012) and rice (IRGSP-1.0; Kawahara et al. 2013, Sakai et al. 2013) (Fig. 1, Supplementary Fig. S1). On the other hand, in ITAG2.4, UTRs do not appear in higher proportions of transcripts: no 5′-UTR in 22,870 transcripts (approximately 66%) and no 3′-UTR in 20,403 transcripts (approx. 59%) (Fig. 1, Supplementary Fig. S1). Our high-quality approach to genome annotation assists in understanding the genome functions more precisely. In particular, the information on the newly detected 5′-UTRs facilitates the comprehensive analysis of transcriptional regulation, such as mining of cis-regulatory elements in upstream regions of transcription start sites.

The lengths of open reading frames (ORFs), exons, introns, genic regions and intergenic regions were compared with those in previous genome annotations. In terms of ORFs, transcripts encoding a polypeptide shorter than 100 amino acids went almost unpredicted in this study due to a condition used in ORF prediction (Supplementary Fig. S2, Materials and Methods). The distribution of exon and intron lengths obtained from this study showed a higher proportion in the short range (≤100 bp) in comparison with Arabidopsis (TAIR10), rice (IRGSP-1.0), and ITAG2.4 (Supplementary Fig. S3). The distribution of lengths of genic regions in this study was different from that in ITAG2.4. In our results, the frequency of short genic regions (≤1500 bp) was lower and the frequency of long genic regions and (>18,000 bp) was higher than in ITAG2.4 (Supplementary Fig. S4A). On the other hand, the relative frequency of the lengths of intergenic regions was similar to that in ITAG2.4 (Supplementary Fig. S4B).

**Fig. 2:**
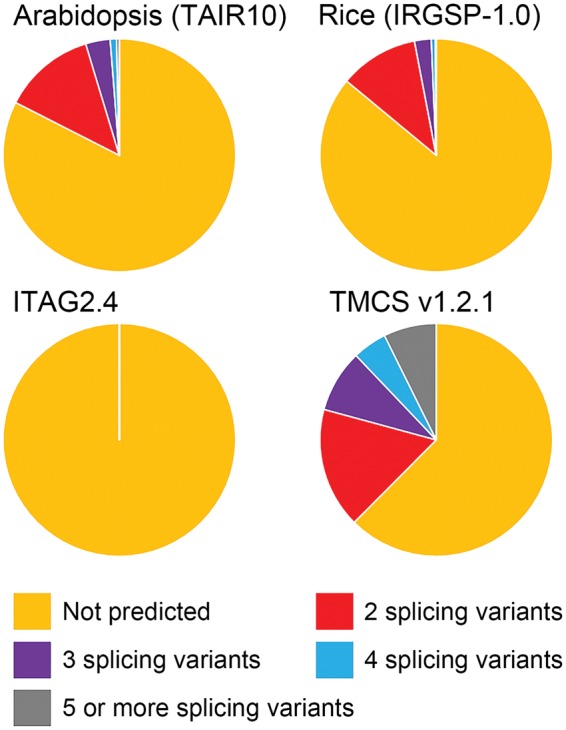
Gene loci with predicted splicing variants. Classification of loci according to the number of splicing variants for each locus in the genomes of Arabidopsis (TAIR10), rice (IRGSP-1.0) and tomato (ITAG2.4 and TMCS v1.2.1).

We found that 10,910 loci (approximately 40% of all loci) proposed two or more splicing variants in TMCS v1.2.1, while ITAG2.4 describes a single transcript (no variants) at each locus (Fig. 2). The ratio of loci with two or more splicing variants was higher than for Arabidopsis (TAIR10) and rice (IRGSP-1.0). In a manual validation, we could find many transcript variants of TMCS v1.2.1 that are consistent with ESTs, and not used in the prediction of gene structure. For example, among three transcript variants predicted on TMCS01g1004430 locus, a new intron was predicted in TMCS01g1004430-01 (Supplementary Fig. S5). An EST in which the intron was spliced out and two ESTs in which the intron was retained were both found, suggesting that this alternative splicing event actually occurs (Supplementary Fig. S5). We also found an example showing an alternative splicing event conserved with Arabidopsis. An Arabidopsis E3 ligase XBAT35 (AT3G23280) has two isoforms caused by skipping the eighth exon (Carvalho et al. 2012). Since the eighth exon encodes a nuclear localization signal (NLS), the alternative splicing event controls subcellular localization of the XBAT53 protein. A tomato gene orthologous to the XBAT35 gene is Solyc09g090160.2 in ITAG2.4 and TMCS09g1008670 in TMCS v1.2.1. Whereas Solyc09g090160.2 has a single transcript retaining the eighth exon, TMCS09g1008670 has four splicing variants: the eighth exon spliced out in TMCS09g1008670-01 and TMCS09g1008670-04 but retained in TMCS09g1008670-02 and TMCS09g1008670-03 (Supplementary Fig. S6A). As in the case with Arabidopsis XBAT35, the eighth exon of the tomato gene is corresponding to the NLS peptide (Supplementary Fig. S6B). These results indicate that the splicing variants predicted in this study contain true variants. Although redundant variants and artifacts are also probably contained, we are going to solve them by improving the procedure for prediction of gene structures and by manual curation in future updates.

#### Functional annotation of TMCS transcripts

Each transcript predicted in TMCS v1.2.1 was functionally annotated. The functional annotations include GO terms (Gene Ontology Consortium 2015), KEGG Orthology (KO) terms (Kanehisa 2016), protein families and domains, and results of BLAST searches. The GO terms protein families and domains needed assigning by the InterProScan tool (Jones et al. 2014); KO terms needed assigning by the KEGG Automatic Annotation Server (KAAS; Moriya et al. 2007). The sequence databases used in the BLAST searches were the NCBI non-redundant protein sequences (nr) (O’Leary et al. 2016), UniProtKB/Swiss-Prot (UniProt Consortium 2015), TAIR10 protein sequences (Lamesch et al. 2012), IRSGP-1.0 protein sequences (Kawahara et al. 2013, Sakai et al. 2013), and potato (PGSC) protein sequences (Potato Genome Sequencing Consortium et al. 2011). In functional annotation with GO terms, 824 terms in biological process, 977 terms in molecular function, and 248 terms in cellular components needed assigning to 35,879 transcripts. In particular, 119 of the 415 new genes (Supplementary Table S2) were assigned with 18 GO terms in biological process, 33 GO terms in molecular function, and four terms in cellular components (Table 1). InterProScan analysis allowed annotation of 19,047 transcripts with 3,200 protein families and 33,145 transcripts with 3,098 domains. Analysis with the KAAS assigned 3,422 KO terms to 21,859 transcripts. The top three KO terms assigned to the most transcripts were myb protein (K09422 to 188 transcripts), peroxidase (K00430 to 138 transcripts) and multidrug resistance protein (K03327 to 127 transcripts). The BLAST searches allowed annotation of 53,911, 44,052, 50,480, 49,115, and 51,594 transcripts with the nr, UniProtKB/Swiss-Prot, TAIR10, IRSGP-1.0, and PGSC databases, respectively. Thus, the functional annotation based on the BLAST search with the nr showed the highest coverage (98.4% of all transcripts) compared with other methods.

**Table 1:** Gene Ontology (GO) terms assigned to genes predicted in TMCS v1.2.1 but not in ITAG2.4 or Micro-Tom HTCs

GO IDs	GO terms	Numbers of loci
*Biological process*		
GO:0055114	Oxidation-reduction process	18
GO:0055085	Transmembrane transport	8
GO:0015074	DNA integration	5
GO:0008152	Metabolic process	3
GO:0006508	Proteolysis	3
GO:0006468	Protein phosphorylation	3
GO:0019856	Pyrimidine nucleobase biosynthetic process	2
GO:0006836	Neurotransmitter transport	2
GO:0042891	Antibiotic transport	1
GO:0032328	Alanine transport	1
GO:0030001	Metal ion transport	1
GO:0019752	Carboxylic acid metabolic process	1
GO:0009607	Response to biotic stimulus	1
GO:0006869	Lipid transport	1
GO:0006511	Ubiquitin-dependent protein catabolic process	1
GO:0006397	mRNA processing	1
GO:0003333	Amino acid transmembrane transport	1
GO:0001822	Kidney development	1
*Molecular function*		
GO:0003676	Nucleic acid binding	31
GO:0020037	Heme binding	18
GO:0016705	Oxidoreductase activity, acting on paired donors, with incorporation or reduction of molecular oxygen	18
GO:0005506	Iron ion binding	18
GO:0008270	Zinc ion binding	15
GO:0046983	Protein dimerization activity	9
GO:0003677	DNA binding	6
GO:0046872	Metal ion binding	5
GO:0005524	ATP binding	5
GO:0016812	Hydrolase activity, acting on carbon-nitrogen (but not peptide) bonds, in cyclic amides	2
GO:0016787	Hydrolase activity	2
GO:0016758	Transferase activity, transferring hexosyl groups	2
GO:0016747	Transferase activity, transferring acyl groups other than amino-acyl groups	2
GO:0015293	Symporter activity	2
GO:0005328	Neurotransmitter:sodium symporter activity	2
GO:0004190	Aspartic-type endopeptidase activity	2
GO:0004151	Dihydroorotase activity	2
GO:0003824	Catalytic activity	2
GO:0043565	Sequence-specific DNA binding	1
GO:0043531	ADP binding	1
GO:0036459	Thiol-dependent ubiquitinyl hydrolase activity	1
GO:0030170	Pyridoxal phosphate binding	1
GO:0016831	Carboxy-lyase activity	1
GO:0015655	Alanine:sodium symporter activity	1
GO:0008757	S-adenosylmethionine-dependent methyltransferase activity	1
GO:0008289	Lipid binding	1
GO:0005509	Calcium ion binding	1
GO:0005488	Binding	1
GO:0004743	Pyruvate kinase activity	1
GO:0004497	Monooxygenase activity	1
GO:0004222	Metalloendopeptidase activity	1
GO:0003723	RNA binding	1
GO:0003690	Double-stranded DNA binding	1
*Cellular component*		
GO:0016021	Integral component of membrane	10
GO:0016020	Membrane	9
GO:0005634	Nucleus	1
GO:0005622	Intracellular	1

In the BLAST search with the nr database, no homologous protein appeared for any transcripts of 96 of the 415 novel genes. In addition, 192 of the 415 novel genes needed annotating as ‘uncharacterized protein’, ‘hypothetical protein’ or ‘unnamed protein’ (Supplementary Table S2). Thus, functional annotations for the 288 novel genes remained unclear after the BLAST search. The InterProScan analysis assigned descriptions about a protein domain or family on only 87 of the 288 genes. Among the 87 genes, 38 needed annotating with transposon-related domains or family such as ‘Ribonuclease H-like domain’ and ‘Transposon, En/Spm-like’.

#### Construction of locus groups

Easy access to the information on correspondence among ESTs, HTCs, and transcripts in ITAG2.4 and TMCS v1.2.1 is important for streamlined identification of experimental resources of interest. To that end, these transcript sequences needed classifying into a single group based on both sequence homology and genomic position. The groups were designated with the nomenclature ‘locus group ID’, such as LG0005554. It needs noting that each locus group included transcripts from one or more loci in ITAG2.4 or TMCS v1.2.1 due to differences in the methods used for gene structure analysis for TMCS v1.2.1 and ITAG2.4.

### Integration of genetic information

Genomic variations including induced and spontaneous mutations need utilizing as powerful genetic tools to understand gene functions. Access to information on the positional relationship between genetic variations and predicted gene models is part of TOMATOMICS.

#### Mapping of flanking sequences of T-DNA insertion sites

Of the 69 flanking sequences of the T-DNA insertion sites of T-DNA tagged lines provided through the TOMATOMA website (Shikata et al. 2016), 62 were mapped onto the reference genome sequences. The mapping results suggest that 34, 24, and 21 lines respectively harbor a T-DNA insertion in an intergenic region, an exon, and an intron (Supplementary Table S3).

#### SNP and InDel calling

Genetic variations, namely, SNPs, insertions and deletions (InDels) in the Micro-Tom genome relative to the Heinz 1706 genome were previously identified by mapping short reads of Micro-Tom onto the Heinz 1706 reference genome sequence build 2.4 (SL2.40) (Kobayashi et al. 2014). However, since the Heinz 1706 genome sequence became updated to build 2.5 (SL2.50), these genetic variations should be re-analyzed with the new genome sequence. Therefore, by mapping the short reads of Micro-Tom onto the SL2.50, we called SNPs and InDels between the genomes of the two cultivars, resulted in 1,692,098 SNPs, 143,518 insertions, and 92,137 deletions identified. Information on the genetic variations identified in this study was stored in the TOMATOMICS database.

### Content in the TOMATOMICS database

In order to furnish seamless access to integrated genetic and genomic information, TOMATOMICS implemented strong search functions and a genome browser. Through these functionalities, detailed information on genes such as functional annotation, genomic position, genetic variations, expression profiles, and gene expression networks appeared with a few clicks.

#### Search functions

**Fig. 3:**
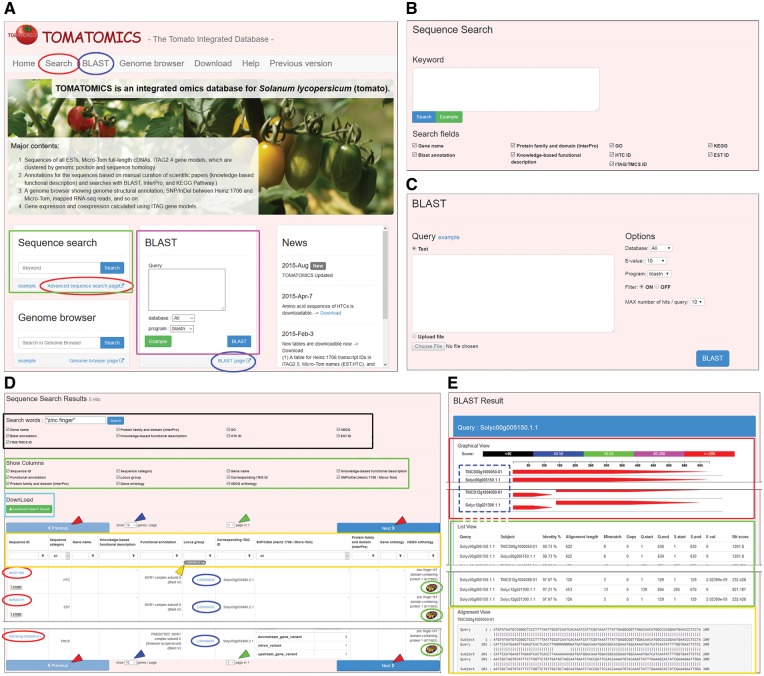
Search functions. A. The top page of TOMATOMICS. The green box and purple box indicate forms for keyword search (labeled ‘Sequence search’) and BLAST search functions, respectively. Red and blue ovals indicate links to advanced search pages for keyword search and BLAST search functions, respectively. B. An advanced search page for keyword searches. Types of information targeted in the search are selectable from checkboxes listed below the text area. C. An advanced BLAST search page. Search queries can be submitted by pasting text or uploading a file in the left panel. Options are set in the right panel. D. A page showing a result of a keyword search in a table. A new search with keywords can be submitted using the form indicated by the black box. The retrieved information is shown in the table format. The columns or fields to be shown in the table are selectable using the checkboxes (shown within the green box). The retrieved information is downloadable by the green button (shown within the cyan box). Each column has filter functions (yellow box) with an arbitrary keyword to selectively display retrieved records, such as rows, genes, or loci. The keyword used for the filtering is displayed at the top of a column (yellow arrowhead). Red and blue ovals indicate hyperlinks to web pages providing detailed information on sequences and locus groups, respectively. Blue and green arrowheads indicate selectors to set the maximum number of rows in the table and to jump between pages, respectively. Red arrowheads indicate the buttons to show a previous or next page of the table. E. A page showing the results of a BLAST search. The results are displayed in a graphical representation (Graphical View, red box), in a table (List View, green box) and as a sequence alignment (Alignment View, yellow box). Sequence IDs in the Graphical View (broken blue box) provide links to pages with detailed information on the sequences.

To access sequences and their associated information mentioned above, keyword search and homology search functions are available in TOMATOMICS. For the user’s convenience, the two search functions appear on the top page with simple search forms, plus on a page specialized for each search function (Fig. 3A, B, C). The keyword search function, labeled ‘Sequence search’, searches the sequences of ESTs, HTCs, and ITAG and TMCS transcripts with annotations containing one or more submitted keywords (queries). The keywords appear as independent queries when the keywords are separate using a space. When multiple words are enclosed in quotation marks (e.g., ‘transcription factor’), a phrase search is performed. In the advanced sequence search page, one or more search fields in which the query keywords are applied are selectable using checkboxes (Fig. 3B). The selectable fields are gene name, protein family and domain, GO term, KEGG orthology, BLAST-based annotations, knowledge-based functional description, and identifiers for sequences; the list of retrieved sequences (records) using the Sequence Search function appears in a table (Fig. 3D). The table shows IDs of retrieved sequences and functional annotations. The table also provides hyperlinks to detailed information pages for each sequence and locus group, as well as hyperlinks to an external database for access to the primary information. To narrow down the search results in the table, a filter function for each column is enabled (Fig. 3D).

For the homology search function, use was made of the BLAST program (Camacho et al. 2009). A query needs submitting by pasting text or uploading a file in single FASTA or multi-FASTA format (Fig. 3C). Before executing a BLAST search by clicking the ‘BLAST’ button, the BLAST search option parameters need selecting: EST, HTC, ITAG, TMCS, or ALL as the nucleotide database; the threshold e-value; the blastn, tblastn or blastx program; whether to activate the filter option; and the maximum number of retrieved sequences shown per query (Fig. 3C). The search result displayed on a result page comprises three parts: ‘Graphical view’, ‘List view’, and ‘Alignment view’ (Fig. 3E). The Graphical view presents a brief graphical view of the BLAST results by a color chart (Fig. 3E). Each ID of a retrieved sequence joins a detailed information page for the sequence (Fig. 3E). The List view is a table showing detailed information on the alignment by the BLAST program, such as identity, alignment length, mismatches, gaps, e-value, and bit score (Fig. 3E). The Alignment view shows the alignment of query and subject sequences (Fig. 3E).

#### The JBrowse Genome Browser

**Fig. 4:**
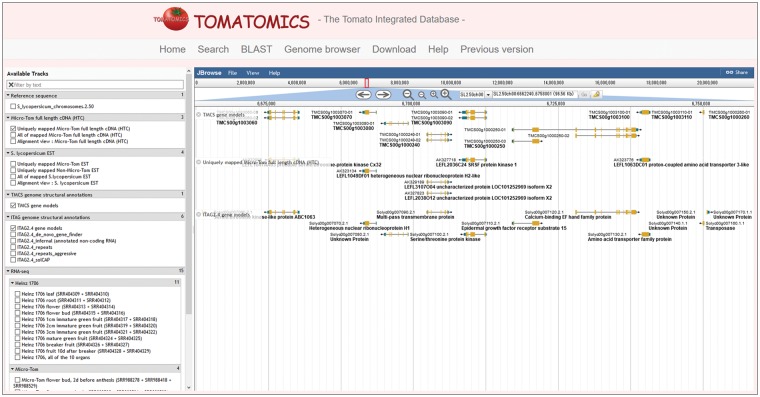
JBrowse genome browser page. The track selector (left panel), menu bar (head of right panel), location bar (below the menu bar), navigation bar (below the location bar) and tracks (main panel on left side) are available for fast, smooth exploration of the genome.

To facilitate comparison of genome annotations, transcripts, genomic variations between Heinz 1706 and Micro-Tom, and T-DNA insertion sites, all of this information was integrated and can be visualized in a JBrowse-based genome browser (Buels et al. 2016). The HTCs, ESTs, TMCS gene models, ITAG gene structural annotations, T-DNA flanking sequences, SGN unigenes, RNA-seq reads, and the SNPs and InDels between Heinz 1706 and Micro-Tom are stored in the genome browser. Tracks displayed need selecting using the track selector (Fig. 4, left-hand pane). Each symbol for transcripts (HTCs, ESTs, TMCS gene models, or ITAG2.4 gene models) on tracks in JBrowse is hyperlinked to a detailed information page. The symbols for T-DNA flanking sequences and SGN unigenes link externally to the original page in TOMATOMA and SGN, respectively. A search function by ID strings of ESTs, HTCs, TMCS loci, ITAG2.4 loci, SGN unigenes, and T-DNA flanking sequences is available as a search box on the navigation bar. This search function also accepts queries for a genomic region represented in a format like ‘SL2.50ch10:61548199..61552895’, which represents the region from position 61,548,199 to 61,552,895 on chromosome 10 of genome SL2.50.

#### Detailed information on locus groups and transcripts

To provide detailed information on locus group and genes, TOMATOMICS has detailed information pages for each locus group, TMCS transcript, ITAG2.4 transcript, HTC, and EST.

**Fig. 5:**
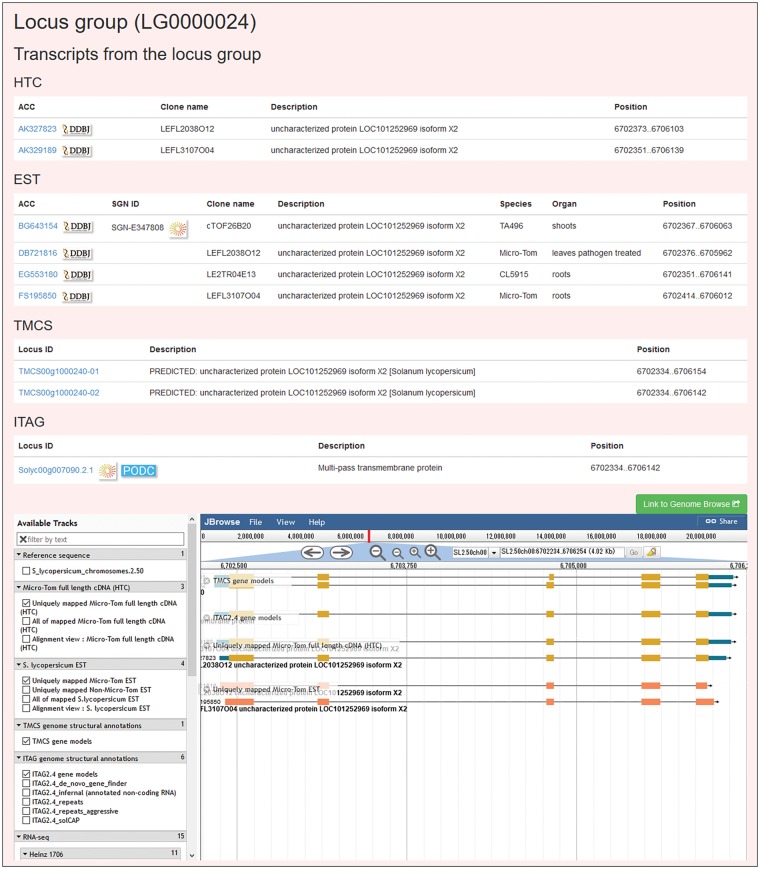
A locus group page. Transcriptomes (HTCs, ESTs, and transcripts predicted in this study and ITAG2.4) inferred to have originated from a single locus are shown on this page. The JBrowse genome browser also shows the genomic positions or regions where the transcripts originated.

The summarized information on each locus group appears on a Locus group page (Fig. 5), which is accessible from a result page of the Sequence Search. Each locus group page displays the locus group ID, a list of transcripts classified in the locus group, descriptions and genomic positions of each transcript (Fig. 5). Additionally, a cDNA clone name displays for HTCs and ESTs. For ESTs, the information on the SGN unigene ID, species, and organ appears. Genomic positions of transcripts appear visually with the JBrowse genome browser (Buels et al. 2016) embedded at the bottom of the Locus group page.

**Fig. 6:**
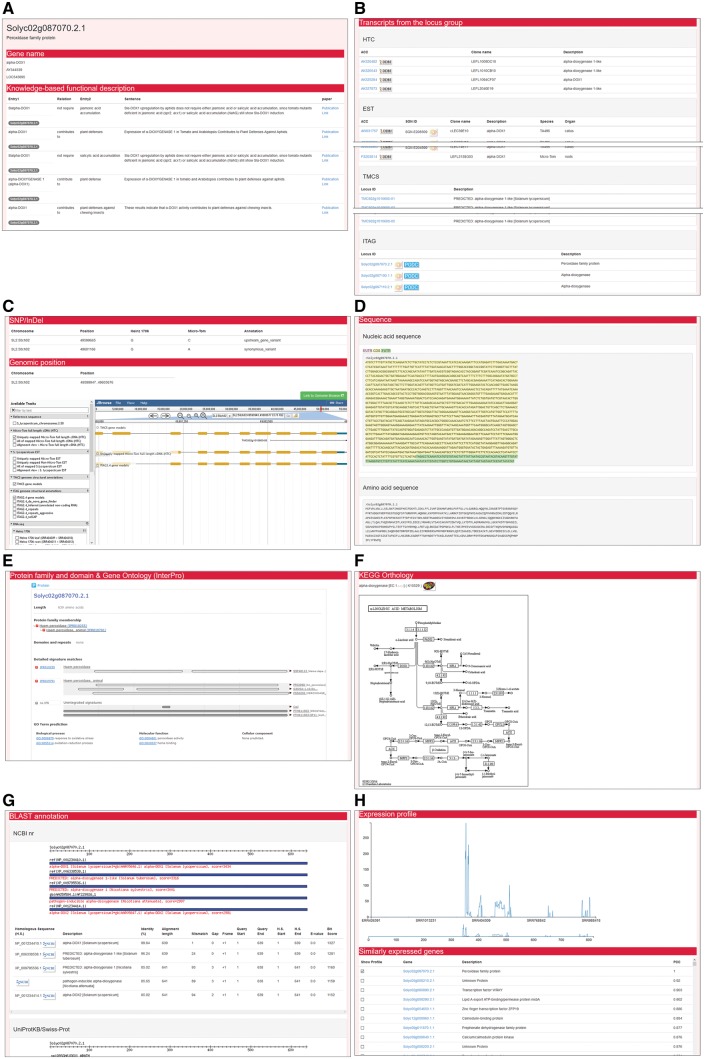
A detailed information page. Each detailed information page contains information on the gene name and knowledge-based functional descriptions (A); transcripts from the locus group (B); SNPs, InDels, and genomic positions with a link to the genome browser (C); nucleotide and amino acid sequences (D); protein families and domains and GO terms (E); KEGG orthology and KEGG pathways (F); the retrieved results from a BLAST search (G); and the expression profile of the transcript and list of genes sharing similar expression profiles (H).

Each detailed information page for transcripts displays gene names, transcripts from each single locus group, SNPs and InDels, genomic positions, nucleotide and amino acid sequences, protein families and functional domains, GO, KO, KEGG pathway information, and BLAST annotations (Fig. 6). In the ‘Knowledge-based functional description’ section, relationships between entries (genes, compounds, and biological phenomenon), a text supporting the relationships, and hyperlinks of the original publications are displayed (Fig. 6A). These knowledge-based descriptions of the transcript are mined from the PubMed papers by natural language processing (NLP) and manual curation as previously described (Ohyanagi et al. 2015). In the ‘Transcripts from the locus group’ section, the identical information to ‘Locus group’ page are displayed (Fig. 6B). In the ‘SNP/INDEL’ section, chromosome names, genomic positions, genotypes in Heinz 1706, genotypes in Micro-Tom, and annotations of variants on or close to the transcript are displayed (Fig. 6C). In the ‘Genomic position’ section, the chromosome name and genomic position of the transcript are shown (Fig. 6C); genomic positions and genomic structures of other transcripts from the locus group and their variants, can be browsed by the embedded JBrowse genome browser. In the ‘Sequence’ section, nucleotide and amino acid sequences of the transcript are displayed in FASTA format (Fig. 6D). In the ‘Protein family and domain and gene ontology’ section, protein families, domains and repeats, and GO terms predicted by InterProScan are displayed (Fig. 6E). In the ‘KEGG orthology’ section, the KEGG orthology and KEGG pathway predicted by KAAS are displayed (Fig. 6F). In the ‘BLAST annotations’ section, results of BLAST searches against the non-redundant protein sequences (nr) provided by NCBI, UniProtKB/Swiss-Prot protein sequences, TAIR10 protein sequences, IRGSP-1.0 protein sequences, and PGSC protein sequences are displayed (Fig. 6G). For the ITAG gene models, knowledge-based functional descriptions, the expression profiles, and similarly expressed genes appear (Fig. 6A, H). In the ‘Expression profile’ section, expression values (in FPKM: fragments per kilobase of exon per million mapped sequence reads) of the transcript in multiple RNA-seq samples collected from SRA are displayed as a plot (Fig. 6H). In the ‘Similarly expressed genes’ section, transcripts showing similar expression profiles to the transcript are listed with Pearson’s correlation coefficient values (Fig. 6H). These FPKM values of the transcripts and the correlation coefficients among them calculate by the mapping of short RNA-seq reads obtained from the SRA database as described previously (Ohyanagi et al. 2015).

## Discussion

TOMATOMICS is a database that not only collects existing information from other databases but also provides value-added information generated by our bioinformatics analysis efforts, serving all of this information through user-friendly web functionality; the collected information is organically linked by mapping analyses and homology analyses, enabling effective cross-searches. Linked information appears summarized on a page and connected via internal hyperlinks for easy browsing. In addition to the internal links, TOMATOMICS provides many external hyperlinks to the primary databases, so is also usable as a portal.

Furthermore, information on gene structural annotation predicted using public RNA-seq data, named as TMCS v1.2.1, provided in TOMATOMICS. In addition to genome-wide improvement in prediction of UTRs and splicing variants, TMCS v1.2.1 includes structural annotation of 415 novel gene loci. One of these 415 loci becomes TMCS01g1015680 annotated as ‘carboxylesterase 1’ based on a BLAST search result (Supplementary Table S2). This gene is actually identical to a previously reported carboxylesterase 1 gene, implicated in human preference for flavor of tomato (Goulet et al. 2012). TMCS05g1019480 and TMCS04g1023010, also predicted in this study, were annotated as ‘adenylate isopentenyltransferase-like’ and ‘zeatin *O*-xylosyltransferase-like’, respectively (Supplementary Table S2). Adenylate isopentenyltransferase is a key enzyme in cytokinin biosynthesis (Kakimoto 2001, Takei et al. 2001), and zeatin *O*-xylosyltransferase which functions in cytokinin metabolism (Turner et al. 1987, Dixon et al. 1989). Cytokinins, a group of plant hormones, have a great impact on crop productivity (Ashikari et al. 2005, Kurakawa et al. 2007, Kudo et al. 2010). In tomato, recent studies are shedding light on positive effects of cytokinins on agricultural traits under saline conditions (Ghanem et al. 2011, Albacete et al. 2014, Aremu et al. 2014). These examples suggest that gene prediction based on RNA-seq data is effective in capturing important genes for biology and agronomy.

Thus far, multiple genetic and genomic information resources have been scattered within the community of tomato biology and breeding. We believe that TOMATOMICS serves as a bridge to connect these multiple resources, including Micro-Tom cDNA clone resources available from NBRP, in a sophisticated fashion and will play a role as a hub for tomato omics biology in the future.

## Materials and Methods

### Design and construction of the database

TOMATOMICS was developed as a web database all function free for use without registration or sign-in. The database was built on a typical client-server system using Linux (CentOS release 5.11, 64-bit) as the operating system, Apache HTTP server (version 2.2.31) as the Web server, MySQL (version 5.0.95) as the relational database management system, PHP (version 5.3.3) for the server-side processing, and JavaScript for the client-side processing. To implement rich user interface applications, JavaScript libraries jQuery (http://jquery.com), jQuery UI (http://jqueryui.com), Bootstrap (http://getbootstrap.com), Vue (http://vuejs.org), Font-awesome (http://fontawesome.io) and D3 (http://d3js.org) were employed. For implementing the genome browser, JBrowse 1.11.6 was installed (Buels et al. 2016).

#### Genome and transcriptome (EST and HTC) sequences from public databases

The genomic sequence (SL2.50) and genomic annotations (ITAG2.4) of Heinz 1706 were downloaded from the SGN database (Fernandez-Pozo et al. 2015). From databases MiBASE (Yano et al. 2006) and KaFTom (Aoki et al. 2010), sequence data of 115,062 ESTs and 13,150 HTCs generated from Micro-Tom were transferred to and stored in TOMATOMICS as their succeeding database. Other tomato ESTs were collected from the NCBI EST database (Boguski et al. 1993). After removing ESTs that were redundant with the ESTs in MiBASE, 185,380 additional ESTs were stored in TOMATOMICS with SGN unigene sequence data also downloaded from the SGN website.

#### Mapping of transcriptome sequences to the reference genome

HTCs and ESTs were mapped to the SL2.50 sequence using the program gmap with default parameters (version 2014-12-29; Wu and Watanabe 2005) and the RNA-seq reads were downloaded from the SRA database (Kodama et al. 2012). Those RNA datasets originated from leaves (SRA accession number: SRR404309, SRR404310), roots (SRR404311, SRR404312), flowers (SRR404313 and SRR404314), flower buds (SRR404315, SRR404316), and fruit at six stages of ripening (SRR404317-SRR404322, SRR404324-SRR404329) of Heinz 1706 and flowers of Micro-Tom at three stages during anthesis (2 days after anthesis: SRR988278, SRR988418, SRR988529; at anthesis: SRR988530-SRR988532; and 4 days after anthesis: SRR988533-SRR988535). The RNA-seq reads were mapped to SL2.50 by the Bowtie2 tool (version 2.2.6; Langmead and Salzberg 2012) and further analyzed by the TopHat2 aligner (version 2.1.0 for Heinz 1706 and version 2.0.13 for Micro-Tom; Kim et al. 2013).

#### Mapping of flanking sequences of T-DNA insertion sites

The flanking sequences of T-DNA insertion lines, which were generated with Micro-Tom as the genetic background and provided from the TOMATOMA database (Shikata et al. 2016), were collected and mapped to the reference genome by the program gmap with default parameters (version 2014-12-29; Wu and Watanabe 2005).

#### SNP and InDel calling

Illumina short reads obtained from genome DNA sequencing of Micro-Tom (accession number DRR000741; Kobayashi et al. 2014) were obtained from the SRA database (Kodama et al. 2012). After quality control by FastQC version 0.11.2 (http://www.bioinformatics.babraham.ac.uk/projects/fastqc/), adapter sequences were trimmed from the reads by the cutadapt tool (Martin 2011) and the low-quality reads filtered out as described previously (Ohyanagi et al. 2015) except the final length of each read was ≥50 bp. The preprocessed reads were aligned to the reference genome sequences of Heinz 1706 (Fernandez-Pozo et al. 2015) with the BWA-backtrack algorithm of the Burrows-Wheeler Aligner version 0.7.12 using default parameters (Li and Durbin 2009). Realignment followed using Genome Analysis Toolkit (GATK) IndelRealigner (version 3.4-46) with default parameters (McKenna et al. 2010, DePristo et al. 2011, Van der Auwera et al. 2013). Duplicated reads, derived from PCR, were identified and marked with the MarkDuplicates feature of Picard tools version 1.136 using default parameters (http://broadinstitute.github.io/picard). Then, SNPs and short InDels were called by the mpileup command in SAMtools (version 1.2) and the call command in BCFtools (version 1.2) with the parameter ‘-f GP,GQ’ for describing genotype quality scores and Phred-scaled genotype posterior probabilities (Li et al. 2009, Li 2011). Low-quality variants with Phred-scaled quality scores lower than 20 were filtered out. Finally, potential effects of the SNPs and InDels on genes such as amino acid replacement and frameshift were predicted by SnpEff program version 4.1 (Cingolani et al. 2012).

#### Gene structure analysis

By mapping the 20 RNA-seq samples of Heinz 1706, 20 BAM files were obtained and using each of these BAM files the structure of the tomato genome was analyzed with a pipeline employing Cufflinks tools (version 2.2.1) (Trapnell et al. 2011) to generate a gene transfer format (GTF) file. As an assembly guide, genomic annotations of ITGA2.4 and HTCs were merged by the cuffmerge program within Cufflinks and using this guide, the 20 GTF files made from RNA-seq data were merged into a single GTF file by the cuffmerge program.

To deduce ORFs on each transcript predicted in the merged GTF file, the TransDecoder tool (version 3.0.0) (Haas et al. 2013) was utilized based on a typical procedure suggested in the manual for this tool by applying several optional settings. In the first step, searching for long ORF candidates (TransDecoder.LongOrfs), the ‘-p 50’ option was applied. As a threshold length of the peptide encoded by the ORFs, the default setting (100 amino acids) applied. In a use of TransDecoder.Predict program, the ‘–single_best_orf’ option was applied to assign a single ORF to a transcript. In this step, results of a blastp search against the TAIR10 protein database (Lamesch et al. 2012) and a domain search against the Pfam database (version 30.0; Finn et al. 2016) were fed to give priority to the ORFs encoding a polypeptide, which is similar to a known plant protein and/or contains known functional or conserved domains. Each transcript on which an ORF was predicted was adopted for a gene model.

#### Creation of TMCS locus IDs

Based on the gene structures obtained in this study, we created TMCS locus IDs for the tomato genome. A general feature format (GFF) file describing the gene structures of TMCS ver. 1.2.1 was generated using a utility Perl script (cdna_alignment_orf_to_genome_orf.pl) provided on the TransDecoder website (https://transdecoder.github.io/) followed by format modification using an in-house Perl script.

#### Construction of locus group IDs

To integrate information on sequences and IDs provided from external databases as clearly as possible, ESTs, HTCs, ITAG genes, and TMCS genes were grouped based on both sequence homology and genomic position. The resulting groups were designated ‘locus groups’ and assigned with a series of ‘LGXXXXXXX’ IDs, where the Xs are seven digits for the group IDs (e.g. LG0005554).

#### Functional annotation of transcripts

The ESTs, HTCs, and transcripts of TMCS v1.2.1 were annotated using InterProScan (version 5.19-58.0; Quevillon et al. 2005), KAAS (Moriya et al. 2007) and the blastp program (version 2.2.30; Camacho et al. 2009). In the KAAS analysis, databases for all available species (i.e. dicotyledoneae, monocotyledoneae, chlorophyceae and rhodophyceae) were employed. In the BLAST search, the protein databases of UniProtKB (Poux et al. 2014), Arabidopsis (TAIR10; Lamesch et al. 2012), rice (IRGSP-1.0; Kawahara et al. 2013, Sakai et al. 2013) and potato (PGSC version 3.4; Potato Genome Sequencing Consortium et al. 2011) were used. BLAST searches were performed with the following parameters: ‘-evalue 1e-05’ and ‘-max_target_seqs 5’.

## Funding

This work was supported in part by Grants-in-Aid for Scientific Research (No. 24113518 and No. 26113716 to K.Y.) from the Ministry of Education, Culture, Sports, Science, and Technology of Japan (MEXT) and for Scientific Research (B) (No. 16H04873 to Y.K.) from the Japan Society for Promotion of Science (JSPS). Support was also provided by the MEXT-Supported Program for the Strategic Research Foundation at Private Universities (2014–2018), Research Project Grant (A) by Institute of Science and Technology, Meiji University and by Research Funding for the Computational Software Supporting 70 Program from Meiji University to K.Y.

## Supplementary Material

Supplementary Data

## Abbreviations

GO: Gene Ontology
ID: identifier
InDel: insertion and deletion
KO: KEGG Orthology
ORF: open reading
frame; SNP: single nucleotide polymorphism
UTR: untranslated region
