# Supplementary material for: TOMATOMICS: A Web Database for Integrated Omics Information in Tomato
Source: Plant Cell Physiol. 2017 Jan 6;58(1):e8. doi: 10.1093/pcp/pcw207 (PMC5444566; doi:10.1093/pcp/pcw207)
Supplement: Supplementary Data [file pcw207_Supp.zip › suppl_data/pcp-2016-e-00418-File009.pdf]

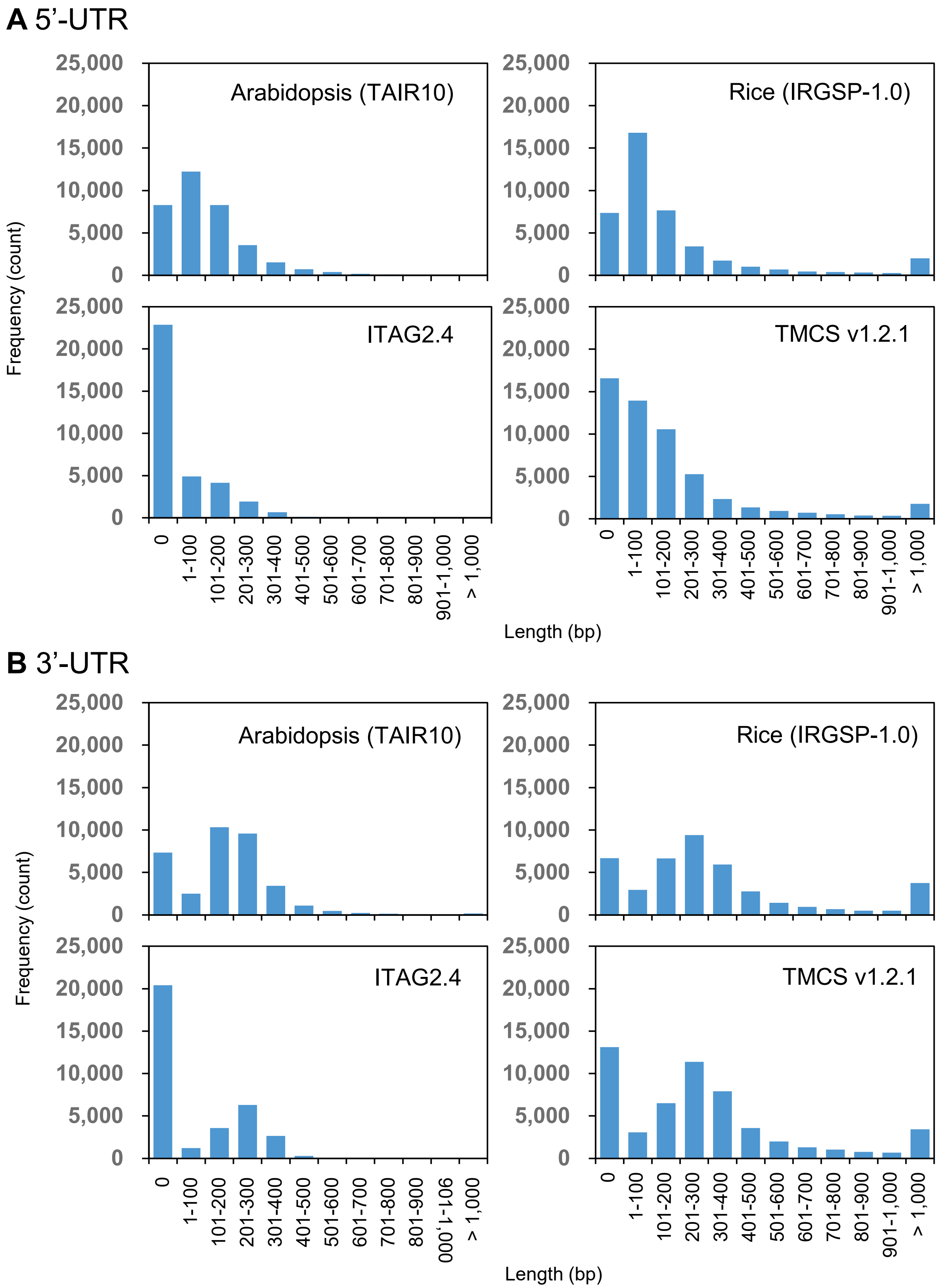

Supplementary Figure S1. Transcripts with predicted 5'-UTRs and 3'-UTRs.
Frequency distributions of 5'-UTR (A) and 3'-UTR (B) lengths in the genomes of Arabidopsis (TAIR10), rice (IRGSP-1.0) and tomato (ITAG2.4 and TMCS v1.2.1) are represented in histograms. The TMCS ver. 1.2.1 is the gene structural annotations obtained from this study.

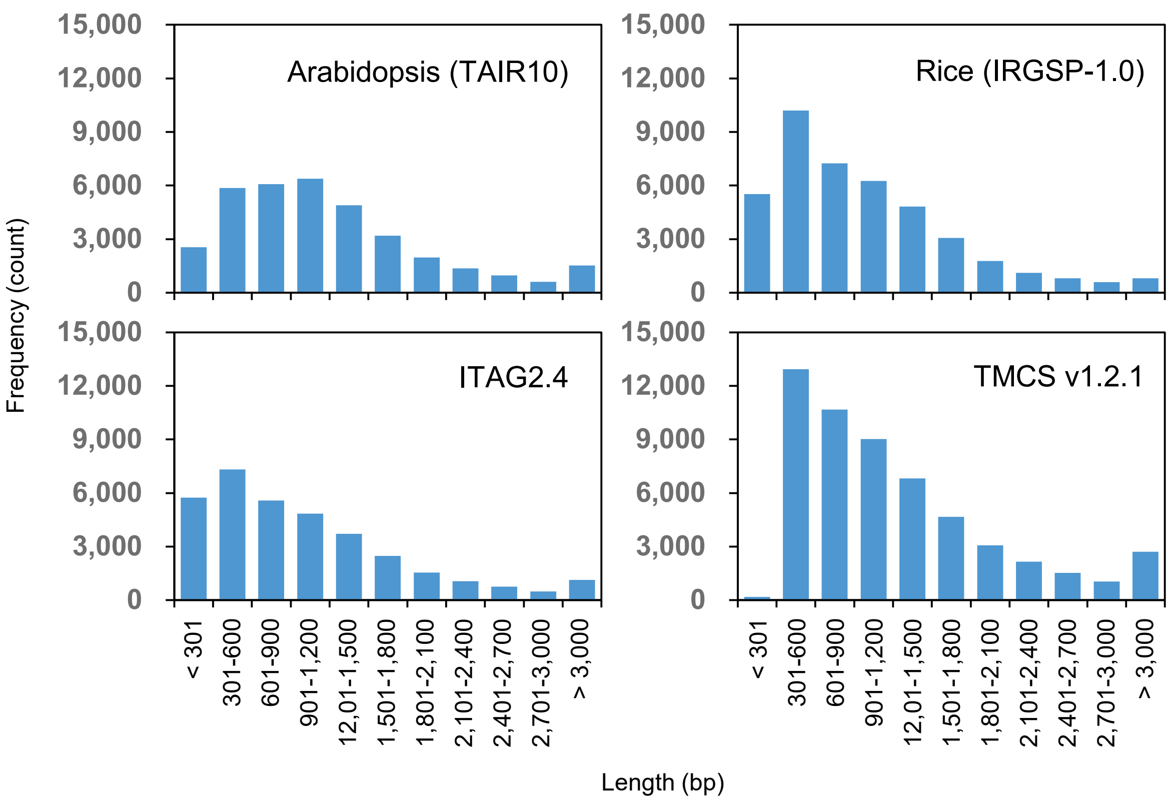

Supplementary Figure S2. Length of predicted open reading frame in transcripts.
Frequency distributions of open reading frame lengths in the genomes of Arabidopsis (TAIR10), rice (IRGSP-1.0) and tomato (ITAG2.4 and TMCS v1.2.1) are represented in histograms. The TMCS ver. 1.2.1 is the gene structural annotations obtained from this study.

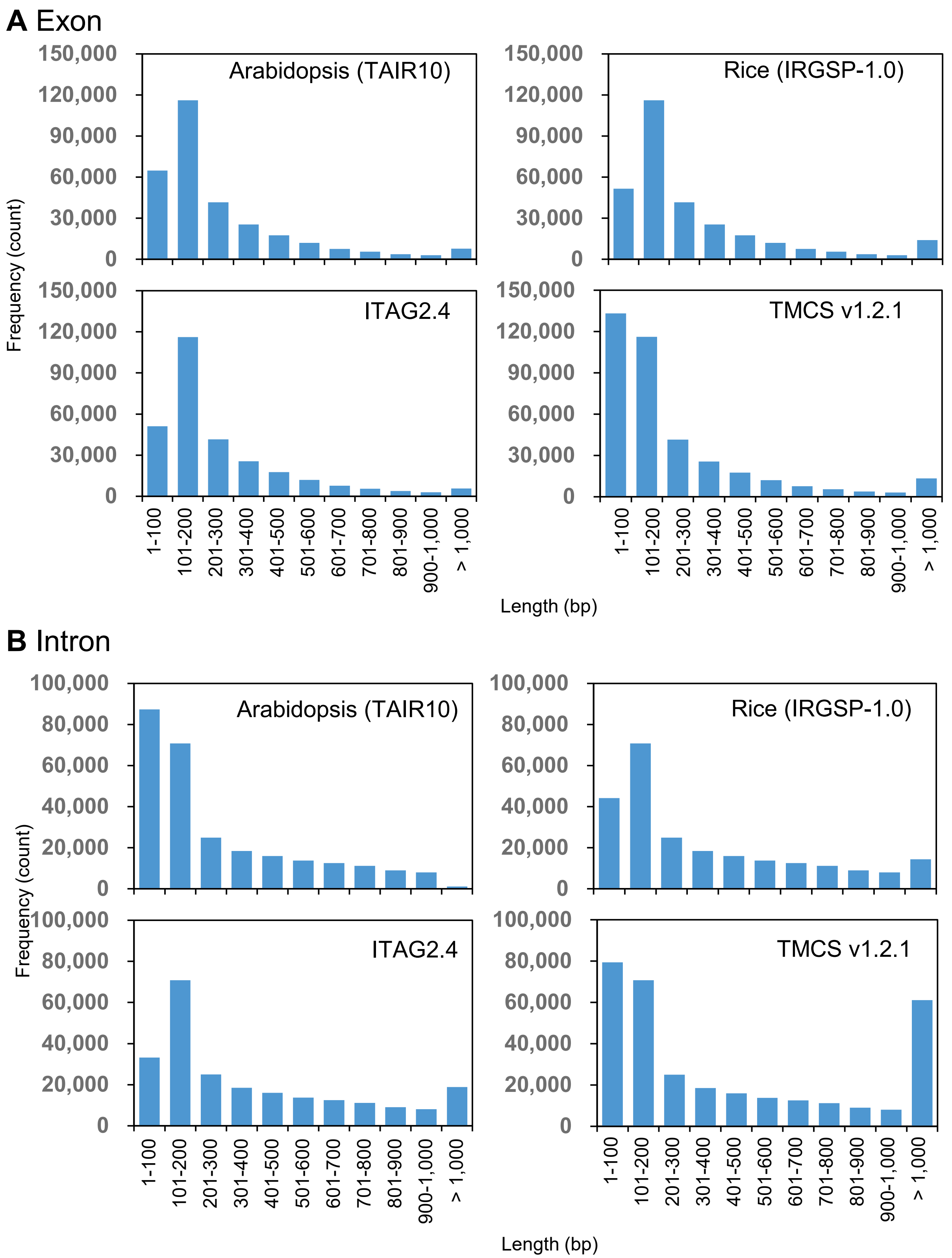

Supplementary Figure S3. Lengths of predicted exons and introns.
Frequency distributions of exon (A) and intron (B) lengths in the genomes of Arabidopsis (TAIR10), rice (IRGSP-1.0) and tomato (ITAG2.4 and TMCS v1.2.1) are represented in histograms. The TMCS ver. 1.2.1 is the gene structural annotations obtained from this study.

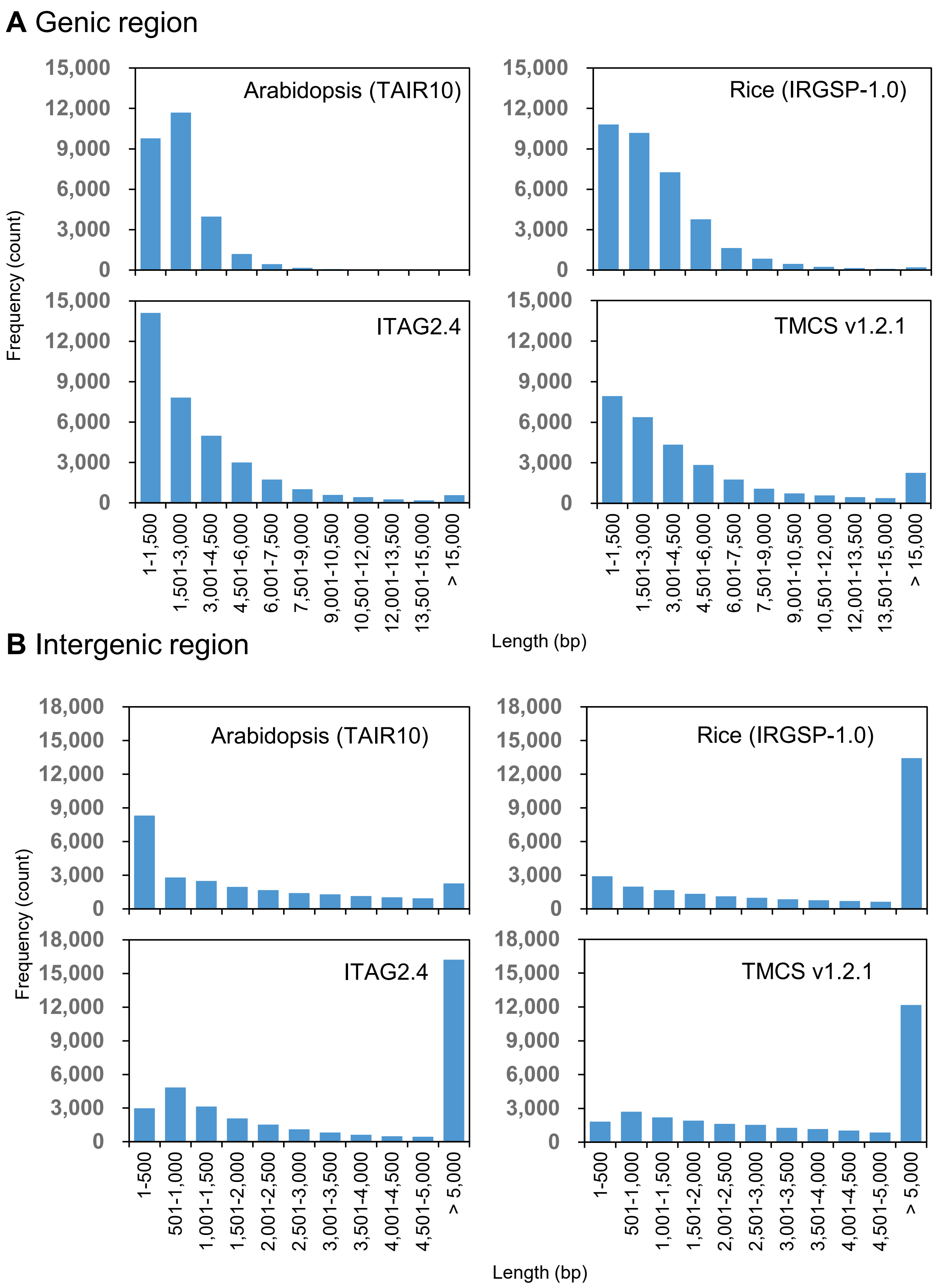

Supplementary Figure S4. Lengths of predicted genic and intergenic regions.
Frequency distributions of genic region (A) and intergenic region (B) lengths in the genomes of Arabidopsis (TAIR10), rice (IRGSP-1.0) and tomato (ITAG2.4 and TMCS v1.2.1) are represented in histograms. The TMCS ver. 1.2.1 is the gene structural annotations obtained from this study.

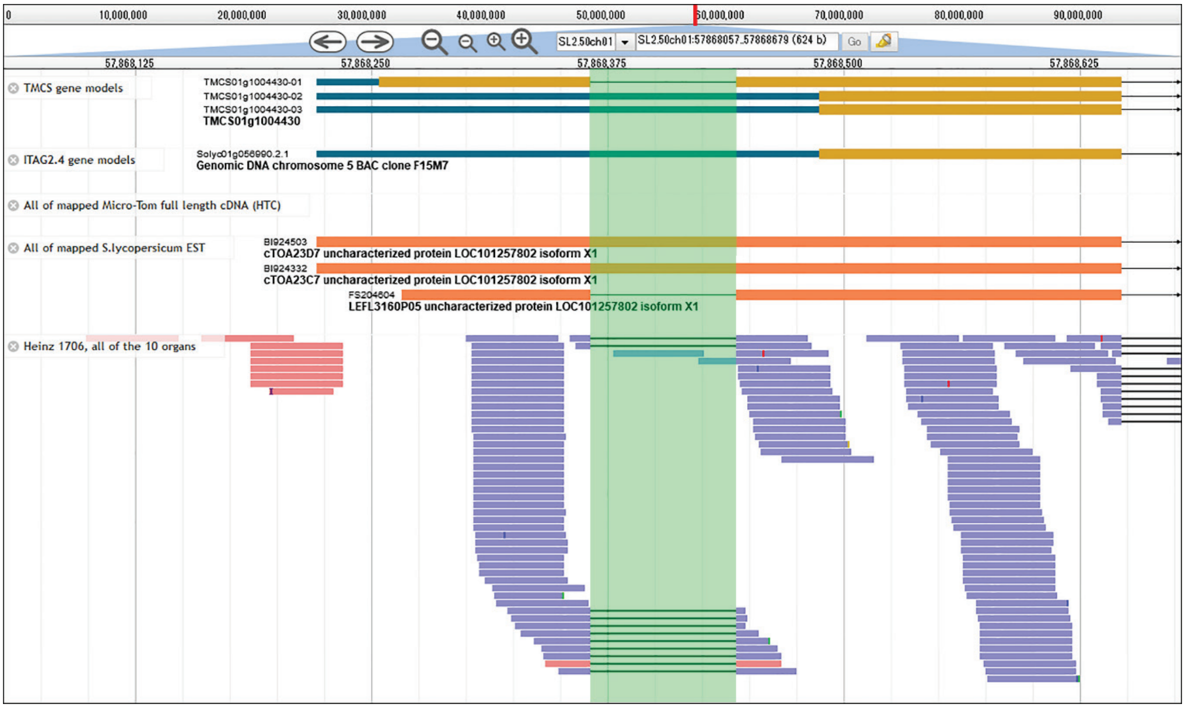

Supplementary Figure S5. An example of splicing variants predicted in TMCS version 1.2.1. Screenshot of JBrowse displaying a genomic region from 57,868,057 to 57,868,679 on chromosome 1 of tomato. Shown tracks are, from upper to bottom, TMCS gene models, ITAG2.4 gene models, all of mapped Micro-Tom full-length cDNA (HTC), all of mapped tomato EST, and RNA-seq reads obtained from 10 different organs of Heinz 1706, as indicated in the left side. Solid lines and boxes represent introns and exons, respectively. In the gene model tracks, brown and blue boxes represent coding sequences and untranslated region, respectively. The region where the intron retention was detected is highlighted in green. This alternative splicing event is supported by ESTs.

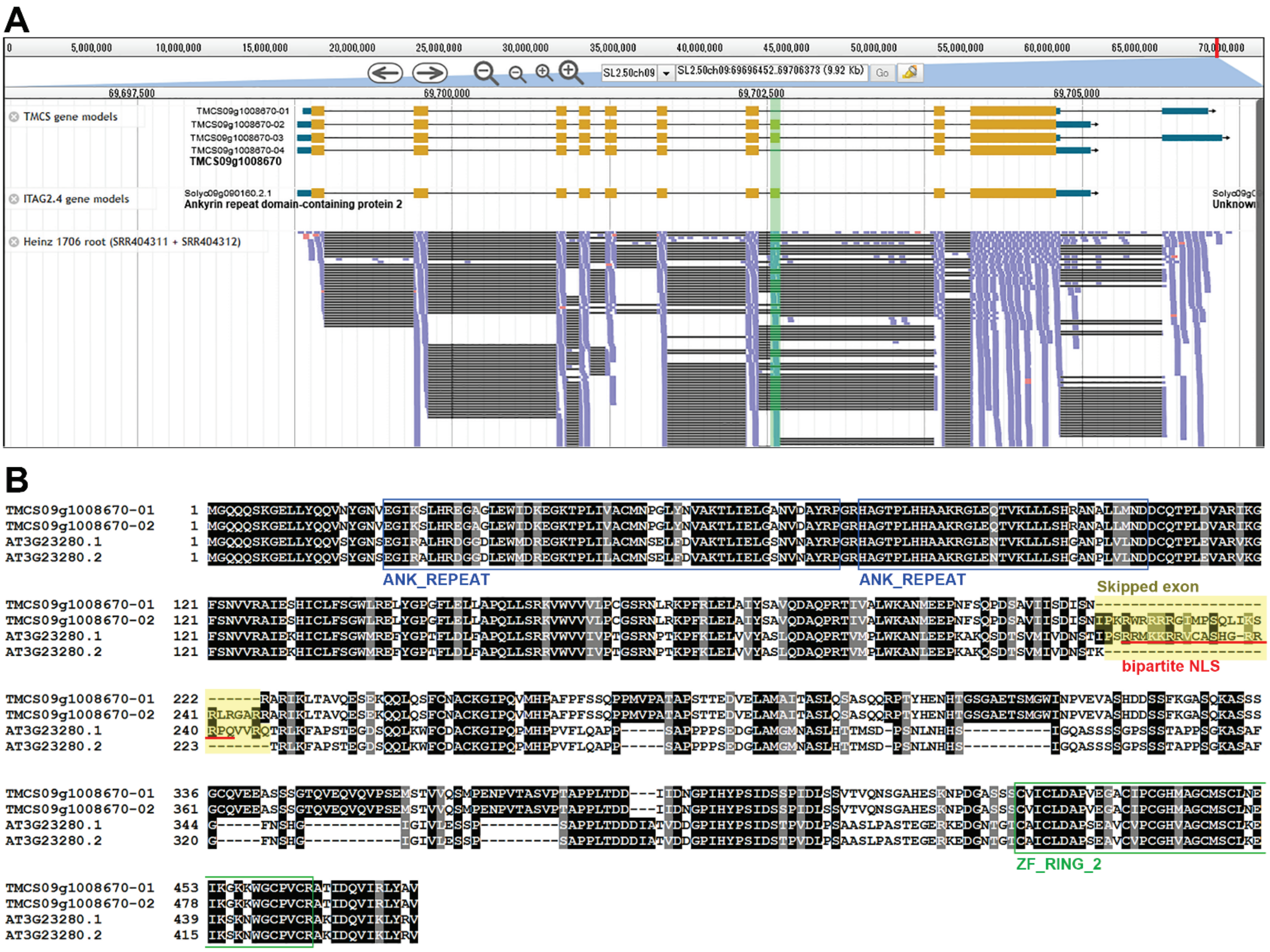

Supplementary Figure S6. An example of alternative splicing event conserved between tomato and Arabidopsis.

A Screenshot of JBrowse displaying a genomic region including a gene locus, TMCS09g1008670 (from 69,696,452 to 69,706,373 on chromosome 19). The 8th exon is skipped in *TMCS09g1008670-01* and *-04* transcript variants. Shown tracks are, from upper to bottom, TMCS gene models, ITAG2.4 gene models, and RNA-seq reads obtained from roots of Heinz 1706, as indicated in the left side. Solid lines and boxes represent introns and exons, respectively. In the gene model tracks, brown and blue boxes represent coding sequences and untranslated region, respectively. The region where the exon skipping was detected is highlighted in green.

B Multiple alignment of the deduced amino acid sequences of splicing variants of the *TMCS09g1008670* and Arabidopsis *XBAT35* (*AT3G23280*). The exon skipping event in *TMCS09g1008670-01* and *AT3G23280.2* results in lacking a nuclear localization signal (NLS) in tomato and Arabidopsis. The alignment was performed using ClustalW version 2.1 (Larkin et al. 2007). Identical and similar residues are shaded in black and grey, respectively. Dashes indicate gaps introduced to maximize sequence similarity. The bipartite NLS reported by Carvalho et al. (2012) is indicated by a red line. The regions encoded by the skipped exon is highlighted in yellow. Ankyrin repeat domains (accession number PS50088) and zinc finger RING domain (PS50089) predicted in the InterProScan analysis are indicated by blue and green boxes, respectively.
